# Concomitant bilateral mandibular cemento-ossifying fibroma and cementoblastoma: case report of an extremely rare occurrence

**DOI:** 10.1186/s12903-021-01794-8

**Published:** 2021-09-07

**Authors:** Madiha Bilal Qureshi, Muhammad Usman Tariq, Jamshid Abdul-Ghafar, Muhammad Raza, Nasir Ud Din

**Affiliations:** 1 Department of Pathology and Laboratory Medicine, Aga Khan University, Karachi, Pakistan; 2 Department of Pathology and Clinical Laboratory, French Medical Institute for Mothers and Children (FMIC), Kabul, Afghanistan

**Keywords:** Cemento-ossifying fibroma, Cementoblastoma, Concomitant

## Abstract

**Background:**

Cemento-ossifying fibroma (COF) and cementoblastoma (CB) are rare benign odontogenic tumors with a predilection for the mandible. Cemento-ossifying fibroma is a fibro-osseous lesion that originates in the tooth bearing areas of jaw and shows cementum-like tissue in a fibrotic stroma. Cementoblastoma is classically related to roots of teeth with the presence of calcified cementum-like material. To date, only a single case of concomitant unilateral COF and CB has been reported in the literature.

**Case presentation:**

We present an unusual case of a 37-year-old female who presented with two discrete bilateral swellings in the right and left mandible for 10 years. The larger tumor involved the left posterior mandible with extension anteriorly to the left and right anterior mandibles, and the smaller tumor was present in right posterior mandible. Radiology revealed two distinct lesions involving both sides of mandible. Histopathological examination showed characteristic features of cemento-ossifying fibroma in sections of the larger tumor and cementoblastoma in sections of smaller tumor.

**Conclusion:**

This case shows the very unique bilateral co-existence of COF and CB, the second case reported in literature to date.

## Background

Cemento-ossifying fibroma (COF) and Cementoblastoma (CB) are two distinct benign tumors of odontogenic origin [1]. Both of these tumors are slow-growing and show affinity for premolar and molar regions of the mandible, followed by the maxilla [2]. COF displays painless expansion of cortical plates of the affected bone, whereas CB presents with sharp toothache-like pain [3]. The incidence of COF peaks in third to fourth decades of life with a female predominance. In contrast, CB has a wide age range with no significant gender disparity [4]. Surgical excision is the treatment of choice. COF can be managed with conservative excision, and recurrence is rare [5]. CB frequently recurs after incomplete excision [6]. Untreated cases of both tumors can show massive enlargement, which may require en-bloc resection [7, 8]. To date, only a single case of concomitant COF and CB has been reported in the literature. Here we present the case of a 37-year-old female who presented with the unique co-existence of concomitant bilateral COF and CB. The patient underwent surgical excision of both lesions.

## Case presentation

**Fig. 1:**
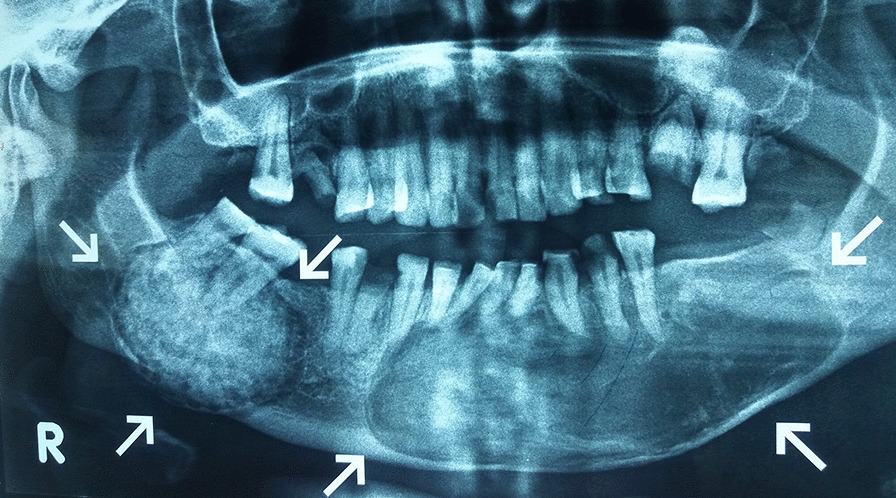
Orthopantomogram showing a well-delineated radiopaque lesion associated with the roots of the right mandibular second and third molars demolishing radiographical details of the roots. Another well-defined radiolucency with focal radiodense areas is visible in the left lower mandible involving roots of the left lower teeth extending till the right canine

A 37-year-old female presented with bilateral mandibular swellings with associated progressive facial asymmetry for 10 years. The swellings gradually increased in size expanding the buccal cortex. She had mild difficulty in mouth opening while movement of temporomandibular joints was normal. The patient developed pain on the posterior aspect of right mandible for one and a half years which became intense with time. There was no history of trauma. Clinical examination revealed a well-demarcated expansile bony swelling in the left lower tooth bearing region extending from the left second premolar to right mandibular canine anteriorly. The swelling was firm and tender with pinkish appearance of mucosa. A clinical diagnosis of ossifying fibroma was considered. Another well-defined painful swelling was present in right posterior premolar area. Orthopantomogram revealed a 7 × 5.5 cm well-defined, expansive radiolucent mass with scattered radiodense calcified areas involving the roots of the first and second left premolars, right and left canines, and right and left central and lateral incisors. The right posterior mandible area showed a 2.5 × 2 cm well-defined radiopaque swelling distorting roots of the second and third molars. There was a visible thin peripheral radiolucent zone surrounding the radiodense area resulting in obliteration and deviation of the roots (Fig. 1).

The patient underwent complete surgical excision with piecemeal resection of both lesions at a peripheral hospital, and the two specimens were sent to our center for processing and primary diagnosis. The larger specimen was labelled as “Left mandibular swelling” and the smaller was labelled as “Right mandibular growth”. The larger specimen contained two grey white soft to firm, multinodular tissue pieces, that measured 7 × 5.5 × 2.5 cm in aggregate. The cut surface was tan-white, lobulated and gritty. The smaller was comprised of three bony hard, tan-white tissue pieces that collectively measured 2.5 × 2 cm.

**Fig. 2:**
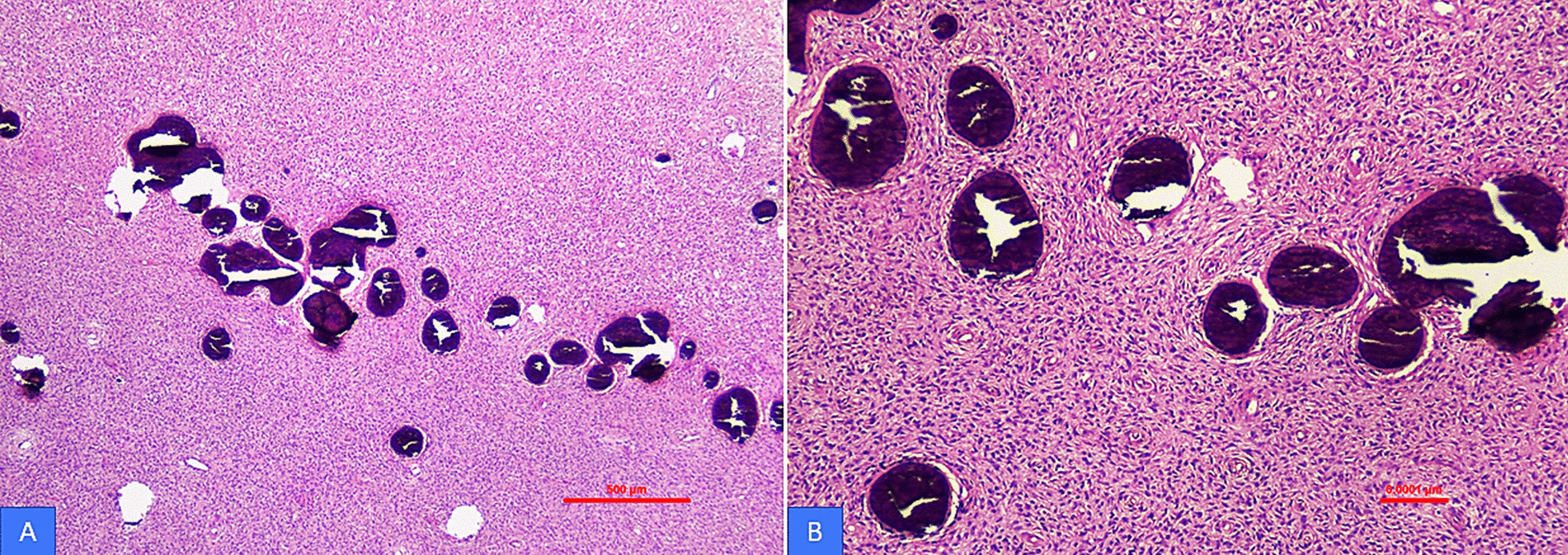
**A**, **B** Fibroosseous proliferation in COF. The osseous component is composed of acellular smoothly outlined rounded structures resembling cementum and present in a bland cellular fibrous stroma (H&E, 40 and ×100 magnification)

**Fig. 3:**
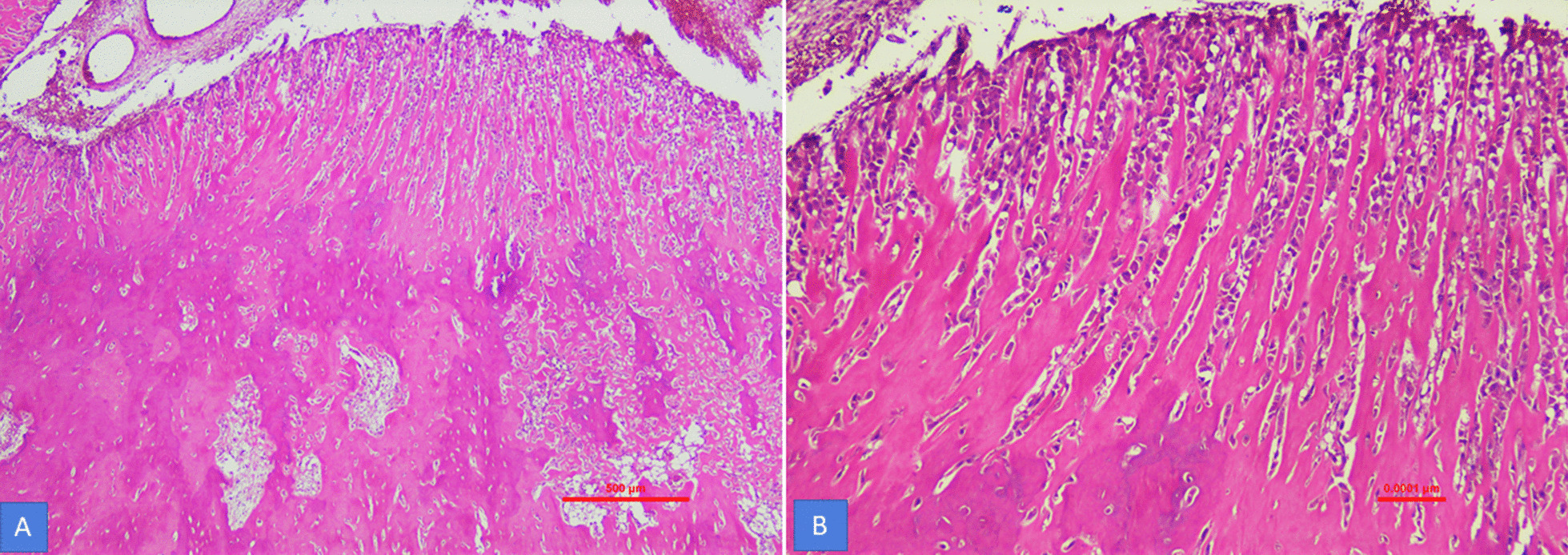
**A** Irregular deposits of woven bone like material with intervening loose fibrovascular stroma and rimming of cementoblasts. **B** Periphery of tumor shows characteristic radiating trabeculae of cementum and parallel cementoblasts (H&E, 40 and ×100 magnification)

Microscopy of the larger specimen demonstrated a fibro-osseous lesion composed of hypercellular fibroblastic stroma with scattered calcified structures. The stromal cells had hyperchromatic nuclei and moderate eosinophilic cytoplasm. No significant atypia or mitosis was present. The calcified material was comprised of variable proportion of basophilic cementum-like tissue and osteoid bone. Curvilinear woven and lamellar bony trabeculae rimmed by osteoblasts were seen (Fig. 2A, B). Histological examination of the smaller specimen revealed a lesion composed of interconnecting thick trabeculae of cementum-like material rimmed by plump cementoblasts in a loose fibrovascular stroma. There was no atypia or pleomorphism (Fig. 3A, B).

Based on all these features, the larger specimen was diagnosed as COF and the smaller specimen as CB. Additional surgical intervention was not required, and it took six months for the patient to regain complete jaw movements. No adjuvant therapy was given. Recurrence was not observed after a follow up of three years. The patient’s consent was obtained for publication. Ethic Review Committee (ERC) exemption was not sought as patient identification was not disclosed in the manuscript.

## Discussion and conclusions

COF and CB are classified as benign mesenchymal odontogenic tumors according to the World Health Organization (WHO) Classification of Head and Neck Tumors [9]. COF is a benign fibro-osseous neoplasm that arises exclusively in the tooth-bearing areas of mandible and maxilla with high affinity for the mandibular premolar and molar area [10]. It is linked to dysregulation of particular micro-RNAs [11]. It typically presents as a painless slow-growing enlargement of the lingual and buccal bony plates. Radiology exhibits a well-demarcated lesion that displays radiolucent and variable radiopaque areas dependent on the duration of lesion. Histologically, COF is an encapsulated lesion comprising of calcified structures in a hypercellular fibrous stroma [12]. The calcified structures are composed of variable amounts of osteoid or bone and basophilic cementum-like tissue. Lesions may demonstrate the presence of curvilinear woven and lamellar bony trabeculae rimmed by osteoblasts. The stromal cells possess hyperchromatic nuclei without significant atypia or mitosis. Conservative surgical excision is curative [13]. Recurrence is extremely low with no sarcomatous transformation reported to date [5, 7].

CB is a benign odontogenic tumor related to the roots of teeth with a predilection for mandibular molars and premolars. It is a rare slow-growing painful lesion of cementum origin that expands the buccal and palatal plates of the involved bone [14]. In this case, the patient had pain in the right posterior side of mandible for more than a year that became sharp with time. Radiology depicts a well-defined radiopaque mass fused with the root of the affected tooth. A characteristic peripheral radiolucent halo is present. Histologically, CB shows thick trabeculae of calcified cementum-like tissue [15]. The cementum appears basophilic with irregular reversal lines mimicking Paget disease of bone. The trabeculae are lined by plump cementoblasts in a loose fibrovascular stroma which may contain occasional osteoclast-like cells. The periphery of the tumor displays uncalcified matrix rimmed with cementoblasts in a radiating pattern. Resection with the affected tooth remains the treatment of choice since the lesion has tendency to recur owing to its unlimited growth potential [16]. In this case, the tooth was not removed but fortunately, there was no recurrence, consistent with reports of similar favorable outcomes of conservative excision [17].

It is very important to diagnose both tumors correctly given the consequential outcomes of these tumors and their histologic mimics. COF most closely resembles ossifying fibroma. Although ossifying fibroma is a benign fibro-osseous lesion of jaw, it still causes progressive and sometimes swift expansion of the involved bone and carries a higher likelihood of recurrence [18]. CB also needs to be clearly distinguished from osteoblastoma and osteosarcoma, two bone forming neoplasms affecting the jaw. The former is categorized as an intermediate tumor with locally aggressive behavior, whereas the latter is a malignant osteoid producing tumor. Osteoblastoma and CB share similar histologic features; however, these entities may be distinguished by the connection of CB to the root of tooth and usual absence of blue-bone common in osteoblastoma [19]. Osteosarcoma also requires definite distinction from CB. The cementoblasts in CB may exhibit a plump appearance with pleomorphism and hyperchromasia seen in osteoblasts of osteosarcoma; however, permeative growth pattern, marked nuclear atypia and frequent mitotic figures are not seen in CB. Radiologic appearances also play a major role in establishing the diagnosis in correlation with histologic findings [20].

COF has been documented to co-exist with other odontogenic tumors including adenomatoid odontogenic tumor, compound odontoma and florid cement-osseous dysplasia [21–23]. In comparison, the co-existence of cementoblastoma with other tumors is extremely rare. Nevertheless, the presence of multiple CB in a single patient has been reported [24]. Concomitant occurrence of COF and CB is exceptionally unusual and, to date, only one such case has been reported in the literature [25]. This patient was a 10-year-old girl with both lesions present in right mandible, which contrasts to the bilateral involvement in our case. Also, the size of COF in the reported case was smaller (3 × 4 cm) in comparison to a larger (7 × 5.5 cm) size in our report. The cause of this co-existence is unknown. COF is related to dysfunction of micro-RNAs, and there is a possibility that some cases of CB also share similar genetic dysregulation that may explain the co-existence; however, no syndromic association has been established yet. More insight into this topic is needed since only limited data is available in the literature.

## Data Availability

Data and materials of this work are available from the corresponding author on request.

## Abbreviations

WHO: World Health Organization
COF: Cemento-ossifying fibroma
CB: Cementoblastoma
ERC: Ethical Review Committee
